# Associations between pulmonary function and cognitive decline in the middle-aged and older adults: evidence from the China Health and Retirement Longitudinal Study

**DOI:** 10.1265/ehpm.22-00158

**Published:** 2022-12-16

**Authors:** Xuefeng Lai, Jian Sun, Bingjie He, Daowei Li, Shengfeng Wang, Siyan Zhan

**Affiliations:** 1 Department of Epidemiology and Biostatistics, School of Public Health, Peking University, 38 Xueyuan Road, Haidian District, Beijing 100191, China; 2 Department of Pulmonary and Critical Care Medicine, Shandong Provincial Hospital, Cheeloo College of Medicine, Shandong University, Jinan 250021, China; 3 Department of Pulmonary and Critical Care Medicine, Shandong Provincial Hospital affiliated to Shandong First Medical University, Jinan 250021, China; 4 Shandong Key Laboratory of Infectious Respiratory Disease, Jinan 250021, China; 5 Research Center of Clinical Epidemiology, Peking University Third Hospital, 49 Huayuan North Road, Haidian District, Beijing 100191, China; 6 Center for Intelligent Public Health, Institute for Artificial Intelligence, Peking University, Beijing 100191, China

**Keywords:** Pulmonary function, Cognitive decline, Middle-aged and older adults, Longitudinal

## Abstract

**Background:**

Studies observing the relationship between pulmonary function and the risk of cognitive impairment in middle-aged and older adults was increasing, but the results were inconsistent. To date, evidence from longitudinal data is scarce and further research is urgently needed.

**Methods:**

We used data from the China Health and Retirement Longitudinal Study. Participants were enrolled in 2011/2013 and followed up in 2013, 2015 and 2018. Pulmonary function was assessed via peak expiratory flow (PEF). Cognitive function, measured by episodic memory and mental status, was assessed through a face-to-face interview in each survey.

**Results:**

A total of 8,274 participants (52.86% males; mean age, 56.44 years) were included. The scores of global cognition (12.46 versus 11.51, *P* < 0.001) of men were significantly higher than women at baseline, with a total of 5096 participants (61.59%) declining during the follow-up. Higher baseline PEF was associated with lower absolute decline in global cognition (OR per 1-SD difference 0.921; *P* = 0.031) and mental status (OR per 1-SD difference 0.9889; *P* = 0.002) during follow-up in men, and significant associations between higher baseline PEF and a lower absolute decline in the episodic memory were both found in men (OR per 1-SD difference 0.907; *P* = 0.006) and women (OR per 1-SD difference 0.915; *P* = 0.022). Second analysis showed that the significant associations between positive PEF variation and a lower rate of 4-year decline in global cognition, mental status and episodic memory were all only found in men. In subgroup analyses, higher PEF at baseline was significantly associated with a lower absolute decline of global cognition among male individuals >60 years. Significant associations between higher PEF at baseline and lower absolute decline in global cognition and episodic memory during follow-up were only found in never-smokers, while higher PEF was related to lower absolute decline in mental status among non-smoking and smoking males.

**Conclusions:**

Pulmonary function correlates with cognitive functions in middle-aged and older people, especially males. Additional studies characterizing early and long-term PEF changes are needed.

**Supplementary information:**

The online version contains supplementary material available at https://doi.org/10.1265/ehpm.22-00158.

## Background

Due to the sustained low birth rate and high-quality medical security, China has entered an aging society. Many Chinese people are currently affected by mild cognitive impairment or cognitive decline, with the prevalence of 5.4% to 25.0% [1]. And the prevalence will continue to rise due to the demographic evolution of including a greater proportion of older adults [2].

Numerous economic, environmental, and biological factors are correlated with cognitive impairment [3–5]. The dynamics of age-related changes in pulmonary function and cognitive function were also emphasized. Several studies have indicated probably predictive effects of pulmonary function on cognitive performance in middle-aged and older adults [6–10]. However, these findings regarding the relationship between pulmonary function and decline in cognition are inconsistent. Reviewing previous studies and literature, a systematic review [11] confirmed that despite substantial apparent support for an accordant association between pulmonary function and cognition in cross-sectional studies, very limited research was found to thoroughly observe their longitudinal associations. Thus, evidence was scarce to substantiate claims of longitudinal relationship between pulmonary function and cognition. In addition, previous studies focused on western populations, and there is little evidence in Asian population [11].

Pulmonary function testing is the most reproducible and objective airflow limitation measurement. This non-invasive test has important guiding significance for the early diagnosis of chronic airway diseases, the judgment of disease severity, the evaluation of medication effect, and the inference of disease prognosis [12]. Peak expiratory flow (PEF) refers to the instantaneous flow rate when the expiratory flow is the fast testing in the process of forced vital capacity measurement. This physiologic measure originally proposed to estimate airflow obstruction, was used to indicate respiratory function in the relevant studies. This study therefore, aims to verify the hypothesis that low PEF could be related to an increased risk of cognitive impairment in community-dwelling middle-aged and older adults, using longitudinal data from the China Health and Retirement Longitudinal Study (CHARLS), which has been collecting a nationally representative sample of Chinese residents aged 45 years and older [13].

## Materials and methods

### Study population

The CHARLS is a nationally representative cohort study of residents aged 45 years or older from 450 communities in China [14, 15]. Samples were chosen from 28 provinces through multistage probability sampling, with the sample stratified by region and within region by urban districts or rural counties firstly. Then 150 county-level units were randomly chosen and 3 primary sampling units were selected within each cunty-level unit, using probability-proportional-to-size sampling technique in both stages [14]. The first national baseline survey for the subject was carried out between June 2011 and March 2012, followed by 3 waves in 2013, 2015, and 2018 [16], with information collected using face-to-face computer-assisted personal interviews. The information of participants was recorded, including sociodemographic information, lifestyle behaviors, and health and cognitive status. Equipment was carried by the reviewers to measure health functioning of the participants [14, 17].

**Fig. 1:**
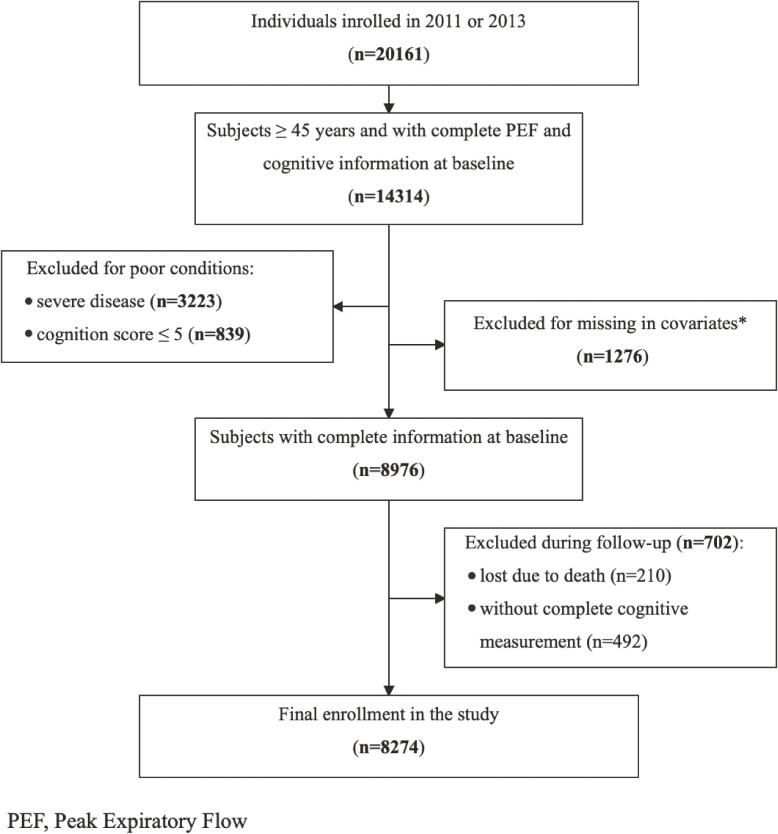
Flowchart of the study subjects in the study *Age: 79 missings; residence: 1 missing; marital status: 1 missing; education: 1096 missings; health insurance: 26 missings; smoking: 418 missings; alcohol use: 7 missings; height: 68 missings; weight: 77 missings; hypertension: 12 missings; diabetes: 26 missings; dyslipidemia: 50 missings

In this study, we included CHARLS participants entered in 2011 and 2013 who were over 45 years old with complete measurement of PEF and cognition function. Subjects without complete information of covariates (n = 1276) and patients suffering from severe diseases or conditions known to affect cognitive function (n = 3223) were excluded (e.g., depression, a history of traumatic brain injury, cerebral infarction, or cerebrovascular disease, malignant tumors). Consistent with a previous study that excluded participants with the 5% lowest cognitive scores, a cut-off of 5 was used [18]. Individuals with a global cognition score of less than or equal to 5 were excluded as the cognition function was too impaired for the participants to complete the cognitive tests or questionnaires (n = 839). During the follow-up, individuals without cognitive scores at any wave were also excluded (n = 702), including 210 subjects who were lost to follow-up because of death. Finally, a complete case approach was used to address missing data for primary analyses (Fig. 1). The survey was approved by the Institutional Review Board (IRB00001052-11015). Written informed consent was provided by all subjects at each study visit.

### Measurement of PEF

PEF is commonly used to check pulmonary ventilation function, which has a good correlation with forced expiratory volume in the first second (FEV_1_) and can better reflect the patency of the airway, measure the function of the large airway and understand the respiratory muscle strength [19]. PEF at each visit was measured using a peak flow meter (EverpureTM, Shanghai, China). Participants were instructed to breathe deeply, place their lips around a disposable mouthpiece, and blow as hard and as fast as possible in a standing position. The interviewers recorded the reading and reset the meter. The next measurement started after waiting 30 seconds with a stopwatch. Three measurements were conducted [14, 20], and the mean value of 3 measurements was calculated for each participant.

### Measurement of cognitive function

Cognition measures of CHARLS included episodic memory and mental status [15, 21], which were conducted with the Word Recall Test (WRT) and the Mental Status Test (MST), respectively: (1) episodic memory was evaluated with WRT by immediate word recall and delayed word recall. After the study personnel read 10 Chinese nouns, respondents were asked to repeat the 10 words in any order as many as they could immediately (immediate word recall) and recall the words 4 min later (delay recall). The word recall score ranged from 0 to 10, determined by the average number of correct words. Construct validity and consistency of WRT were previously confirmed to be good [22]. (2) mental status was assessed with MST including figure drawing and Telephone Interview for Cognitive Status-10 (TICS-10). In the figure drawing, participants were asked to re-draw a formerly shown figure as similarly as possible and received a score of 1 or 0 based on the completeness of the task they finished. TICS-10 is a well-established and valid measure used to screen cognitively impaired elderly which involves ten questions asking respondents to name the date, the day of the week, the season of the year, and serial 7 subtraction from 100 (up to 5 times) [23]. The score was calculated according to the number of correct answers and ranged from 0 to 10. The Chinese version of the above neuropsychological tests have been validated in a subgroup of the CHARLS [24].

Global cognition score was represented by the sum of WRT scores (0 to 10) and MST (0 to 11), ranging from 0 to 21, which showed better cognitive function with a higher score [14]. The measurement was conducted at a face-to-face interview in each wave of the survey. To promote the clinical interpretability of outcome, according to the change of global cognition score, episodic memory, and mental status from the baseline to the last measurement, participants were then divided into two groups: no decline (≥0) and decline (<0) [25].

### Covariate assessment

Based on previous studies [6, 11, 26–28], demographic characteristics included age (years), residence (urban/rural), marital status (married/divorced/single), education (illiterate/primary school/middle school and above) and health insurance status (yes/no). Lifestyle behaviors including smoking status (current/former/never), alcohol use (current/former/never) and social activity (none/inactive/active) were also taken into consideration. Body mass index (BMI) was categorized as underweight (<18.5 kg/m^2^), normal (18.5–23.9 kg/m^2^), overweight (24.0–27.9 kg/m^2^), or obese (>28.0 kg/m^2^) [29]. We also added conditions of chronic disease including diabetes (yes/no), hypertension (yes/no), and dyslipidemia (yes/no). Diabetes was defined as a self-reported physician diagnosis of diabetes, ≥200 mg/dL non-fasting glucose, ≥126 mg/dL fasting glucose, or diabetes medications use [30]. Hypertension was defined as self-reported physician diagnosis of hypertension, measured systolic blood pressure ≥140 mmHg or/and diastolic blood pressure ≥90 mmHg or antihypertensive medications use [31]. Dyslipidemia was determined by self-reported physician diagnosis of dyslipidemia, or total cholesterol ≥240 mg/dl and low-density lipoprotein ≥160 mg/dl, or triglycerides ≥200 mg/dl, or high-density lipoprotein cholesterol <40 mg/dl, or anti-dyslipidemia agents use [19]. Cognition conditions on baseline and years of follow-up were also added as covariates for adjustment.

### Statistical analysis

To identify basic differences between male and female, the chi-squared test for categorical variables and the Student *t*-test for a continuous variable was used. First, we used binary logistic regression models to determine adjusted associations between cognitive decline and per 1-SD higher PEF at baseline in males and females, which were adjusted for baseline age, marital status, education, registered residence, height, weight, smoking status, alcohol use, diabetes, hypertension, dyslipidemia, social activity, health insurance status, and baseline cognitive status. Considering that PEF variation is also an important indicator of respiratory function and the lack of PEF data in 2018, our second analysis calculated the adjusted odds ratio of 4-year cognitive decline for PEF variation from 2011 to 2015, and added baseline PEF into adjustment. The PEF variation was defined as the difference between the PEF in 2015 and the PEF in 2011 divided by the PEF in 2011. Subgroup analyses were also conducted to observe the differences of the association identified above among age and smoking groups. Consistent with previous studies [32], we chose 60 years as a cut-off for subgroup analyses. In the sensitivity analysis, we calculated the E-value to investigate the potential effect of unmeasured confounding [33], and examined associations (1) by defining accelerated cognitive decline as ≥1 SD standardized over follow-up time and age from the mean change on the cognitive score derived from the last tests and baseline; (2) after excluding participants with asthma; (3) using competing risk analysis to estimate the effect of missing follow-up cognitive score on the observed associations.

All tests were two-tailed with a significance level of *P* < 0.05. The OR was reported with its 95% confidence interval (CI) to present the associations. All analyses were conducted using Stata 15.0 (StataCorp LP).

## Results

### Baseline characteristics

A total of 8 274 individuals were included in this analysis, including 4 374 (52.86%) males and 3 900 (47.14%) females. The mean baseline age of males and females was 58.16 (SD: 8.79) years and 56.44 (SD: 8.41) years, respectively. Baseline characteristics of eligible subjects and individuals excluded were shown by sex (Table 1, Supplementary Table 1). Women were more frequently divorced, more likely to be illiterate, overweight or obese, and more living in town (*P* < 0.001). But they were less likely to be regular smokers and regular alcohol drinkers compared with men (*P* < 0.001). The mean PEF in men was 327.57 (SD: 125.98) and that of women was 240.22 (SD: 87.87). Specifically, compared to included subjects, there were more current smoking and drinking in excluded man, and less urban residents in excluded woman.

**Table 1:** Descriptive statistics for eligible individuals at baseline

**Characteristics**	**Overall, N = 8274**	**Male, n = 4374**	**Female, n = 3900**	***P*-value**
Age, mean (SD)	57.35 (8.65)	58.16 (8.79)	56.44 (8.41)	<0.001
Urban, n (%)	3371 (40.74)	1661 (37.97)	1710 (43.85)	<0.001
Marriage, n (%)				
Married	7540 (91.13)	4061 (92.84)	3479 (89.21)	<0.001
Divorced	680 (8.22)	262 (5.99)	418 (10.72)	
Single	54 (0.65)	51 (1.17)	3 (0.08)	
Education, n (%)				
Illiterate	1452 (17.55)	351 (8.02)	1101 (28.23)	<0.001
Primary school	3519 (42.53)	1959 (44.79)	1560 (40.00)	
Middle school	2120 (25.62)	1301 (29.74)	819 (21.00)	
High school and above	1183 (14.30)	763 (17.44)	420 (10.77)	
Smoking, n (%)				
Never	4752 (57.43)	1139 (26.04)	3613 (92.64)	<0.001
Former	790 (9.55)	717 (16.39)	73 (1.87)	
Current	2732 (33.02)	2518 (57.57)	214 (5.49)	
Alcohol use, n (%)				
Never	4557 (55.08)	1303 (29.79)	3254 (83.44)	<0.001
Former	463 (5.60)	395 (9.03)	68 (1.74)	
Current	3254 (39.33)	2676 (61.18)	578 (14.82)	
Health insurance, n (%)	7819 (94.50)	4148 (94.83)	3671 (94.13)	0.160
Socially active, n (%)				
None	4564 (55.16)	2454 (56.10)	2110 (54.10)	0.109
Inactive	2331 (28.17)	1191 (27.23)	1140 (29.23)	
Active	1379 (16.67)	729 (16.67)	650 (16.67)	
Body mass index (kg/m^2^), n (%)				
<18.5	372 (4.50)	209 (4.78)	163 (4.18)	<0.001
18.5∼23.9	4147 (50.12)	2463 (56.31)	1684 (43.18)	
24.0∼27.9	2694 (32.56)	1291 (29.52)	1403 (35.97)	
>28.0	1061 (12.82)	411 (9.40)	650 (16.67)	
Height (m), mean (SD)	1.60 (0.08)	1.65 (0.07)	1.54 (0.06)	<0.001
Weight (kg), mean (SD)	60.77 (10.93)	63.33 (10.98)	57.90 (10.14)	<0.001
Diabetes, n (%)	959 (11.59)	496 (11.34)	463 (11.87)	0.450
Hypertension, n (%)	2914 (35.22)	1535 (35.09)	1379 (35.36)	0.801
Dyslipidemia, n (%)	2172 (26.25)	1120 (25.61)	1052 (26.97)	0.158
PEF (L/min), mean (SD)	286.40 (118.02)	327.57 (125.98)	240.22 (87.87)	<0.001

Abbreviations: PEF, peak expiratory flow.

### Cognitive decline

At baseline, the scores of mental status (8.59 (2.27) versus 7.57 (2.57), *P* < 0.001) and global cognition (12.46 (3.04) versus 11.51 (3.38), *P* < 0.001) of men were significantly higher than women, but women scored better than men in episodic memory (3.94 (1.64) versus 3.87 (1.55), *P* = 0.064). Regarding cognitive decline during baseline to the last wave, there were no significant differences between men and women among absolute cognitive decline scores or groups of no decline and decline. A total of 5096 participants (61.59%; male: 2704, 61.82%; female: 2392, 61.33%) had decline in global cognition during the follow-up, with 4916 participants (59.42%; male: 2608, 59.63%; female: 2308, 59.18%) in mental status and 4145 participants (50.10%; male: 2147, 49.09%; female: 1998, 51.23%) in episodic memory (Table 2).

**Table 2:** Cognitive descriptions of eligible individuals

	**Overall, N = 8274**	**Male, n = 4374**	**Female, n = 3900**	***P*-value**
Cognition at baseline, mean (SD)				
Global cognition	12.01 (3.24)	12.46 (3.04)	11.51 (3.38)	<0.001
Mental status	8.11 (2.47)	8.59 (2.27)	7.57 (2.57)	<0.001
Episodic memory	3.90 (1.60)	3.87 (1.55)	3.94 (1.64)	0.064
Cognition at last wave, mean (SD)				
Global cognition	10.62 (4.18)	11.06 (3.86)	10.13 (4.46)	<0.001
Mental status	6.82 (2.89)	7.26 (2.71)	6.33 (3.01)	<0.001
Episodic memory	3.61 (2.11)	3.61 (2.01)	3.61 (2.22)	0.911
Absolute cognitive decline during baseline to the last wave, mean (SD)				
Global cognition	1.39 (3.63)	1.40 (3.57)	1.37 (3.70)	0.764
Mental status	1.29 (2.74)	1.33 (2.73)	1.24 (2.75)	0.150
Episodic memory	0.29 (2.11)	0.26 (2.04)	0.33 (2.19)	0.130
Follow-up time (years), mean (SD)				
Global cognition	5.92 (1.63)	5.91 (1.63)	5.93 (1.63)	0.580
Mental status	6.17 (1.48)	6.12 (1.51)	6.23 (1.44)	0.001
Episodic memory	6.00 (1.58)	5.97 (1.60)	6.03 (1.57)	0.110
Groups of global cognition change, n (%)				0.650
No decline	3178 (38.41)	1670 (38.18)	1508 (38.67)	
Decline	5096 (61.59)	2704 (61.82)	2392 (61.33)	
Groups of mental status change, n (%)				0.680
No decline	3358 (40.58)	1766 (40.37)	1592 (40.82)	
Decline	4916 (59.42)	2608 (59.63)	2308 (59.18)	
Groups of episodic memory change, n (%)				0.051
No decline	4129 (49.90)	2227 (50.91)	1902 (48.77)	
Decline	4145 (50.10)	2147 (49.09)	1998 (51.23)	

### PEF and cognitive decline

After controlling for explanatory and confounding variables using logistic regression models, higher baseline PEF was associated with a lower absolute decline in global cognition during follow-up in men (OR per 1-SD difference 0.921, 95% CI 0.855, 0.993; *P* = 0.031), whereas no significant association was observed in women. Moreover, significant inverse associations were also found between baseline PEF and absolute decline in the mental status (OR per 1-SD difference 0.889, 95% CI 0.826, 0.956; *P* = 0.002) in men, and similarly, no significant association was found in women. However, significant inverse associations between baseline PEF and absolute decline in the episodic memory were both found in men (OR per 1-SD difference 0.907, 95% CI 0.842, 0.977; *P* = 0.006) and women (OR per 1-SD difference 0.915, 95% CI 0.848, 0.987; *P* = 0.022) (Table 3).

**Table 3:** Odds ratio of cognitive decline for PEF in participants of the CHARLS

	**Global cognition**		**Mental status**		**Episodic memory**	
		
	**OR (95%CI)**	***P*-value**	**OR (95%CI)**	***P*-value**	**OR (95%CI)**	***P*-value**
Unadjusted odds ratio of cognitive decline per 1-SD higher PEF at baseline^†^						
Male (n = 4374)	0.913 (0.859, 0.971)	0.004	0.916 (0.863, 0.974)	0.005	0.850 (0.801, 0.902)	<0.001
Female (n = 3897)	0.948 (0.888, 1.011)	0.101	0.991 (0.929, 1.056)	0.771	0.834 (0.783, 0.889)	<0.001
Adjusted odds ratio of cognitive decline per 1-SD higher PEF at baseline^†^						
Male (n = 4374)	0.921 (0.855, 0.993)	0.031	0.889 (0.826, 0.956)	0.002	0.907 (0.842, 0.977)	0.006
Female (n = 3897)	0.974 (0.904, 1.050)	0.494	0.975 (0.905, 1.051)	0.513	0.915 (0.848, 0.987)	0.022
Unadjusted odds ratio of 4-year cognitive decline for PEF variation between 2015 and 2011^§^						
Male (n = 2815)	0.840 (0.769, 0.918)	<0.001	0.908 (0.839, 0.983)	0.017	0.885 (0.816, 0.959)	0.003
Female (n = 2545)	0.918 (0.851, 0.991)	0.117	0.911 (0.842, 0.987)	0.022	0.989 (0.919, 1.064)	0.770
Adjusted odds ratio of 4-year cognitive decline for PEF variation between 2015 and 2011^§^						
Male (n = 2815)	0.771 (0.683, 0.871)	<0.001	0.905 (0.820, 0.998)	0.046	0.793 (0.706, 0.890)	<0.001
Female (n = 2545)	0.926 (0.841, 1.019)	0.117	0.945 (0.855, 1.045)	0.274	0.952 (0.864, 1.048)	0.314

Abbreviations: OR, odds ratio; CI, confidence interval; PEF, peak expiratory flow.

^†^Adjusted for baseline age, education, marital status, registered residence, height, weight, alcohol use, smoking status, diabetes, hypertension, dyslipidemia, social activity, health insurance status, follow-up time and baseline cognitive status.

^§^Adjusted for baseline age, education, marital status, registered residence, height, weight, alcohol use, smoking status, diabetes, hypertension, dyslipidemia, social activity, health insurance status, baseline cognitive status and baseline peak expiratory flow.

Second analysis that calculated the adjusted odds ratio of 4-year cognitive decline for PEF variation from 2011 to 2015 showed similar results, except for the decline in episodic memory. The significant associations between positive PEF variation and lower rate of 4-year decline in global cognition, mental status and episodic memory were all only found in men (OR per 100% variation 0.771, 95% CI 0.683, 0.871, *P* < 0.001; OR 0.905, 95% CI 0.820, 0.998, *P* = 0.046; OR 0.793, 95% CI 0.706, 0.890, *P* < 0.001), not in women (Table 3).

### Subgroup analyses

As presented in Table 4, subgroup analysis by age showed that higher PEF at baseline was significantly associated with a lower absolute decline of global cognition in man over 60 years (*P* for interaction = 0.009). In analyses stratified by smoking status, significant associations between higher PEF at baseline and lower absolute decline of global cognition and episodic memory during follow-up were only found in individuals who never smoke, while higher PEF was related to a lower absolute decline in mental status among non-smoking and smoking males. However, no statistically significant interactions were noted between the PEF and smoking status at baseline in the fully adjusted models.

**Table 4:** Subgroup analysis of adjusted odds ratio of cognitive decline for PEF at baseline

**Subgroup**	**Global cognition^†^**		**Mental status^†^**		**Episodic memory^†^**	
		
	**Male (n = 4374)**	**Female (n = 3897)**	**Male (n = 4374)**	**Female (n = 3897)**	**Male (n = 4374)**	**Female (n = 3897)**
Age						
45-60 y (n = 5353)	0.985 (0.897, 1.082)	0.975 (0.891, 1.066)	0.901 (0.820, 0.989)*	0.948 (0.866, 1.038)	0.885 (0.804, 0.974)*	0.917 (0.836, 1.005)
>60 y (n = 2921)	0.809 (0.714, 0.916)**	0.962 (0.836, 1.107)	0.865 (0.767, 0.975)*	1.030 (0.896, 1.184)	0.926 (0.822, 1.043)	0.875 (0.763, 1.005)
*P* for Interaction	0.009	0.140	0.778	0.836	0.136	0.674
Smoke						
Never (n = 4749)	0.846 (0.730, 0.981)*	0.965 (0.891, 1.044)	0.848 (0.734, 0.980)*	0.962 (0.889, 1.041)	0.848 (0.733, 0.982)*	0.906 (0.836, 0.982)*
Former or current (n = 3522)	0.955 (0.876, 1.041)	1.088 (0.840, 1.410)	0.907 (0.833, 0.987)*	1.166 (0.890, 1.526)	0.929 (0.852, 1.012)	1.069 (0.816, 1.400)
*P* for Interaction	0.656	0.811	0.230	0.615	0.441	0.553

Legend: Adjusted odds ratio in cognitive decline per 1-SD higher PEF at baseline in participants of the CHARLS according to subgroups of various variables, OR (95%CI).

Abbreviations: OR, odds ratio; CI, confidence interval; PEF, peak expiratory flow.

^†^Adjusted for baseline age, education, marital status, registered residence, height, weight, alcohol use, smoking status, diabetes, hypertension, dyslipidemia, social activity, health insurance status, follow-up time and baseline cognitive status.

*P < 0.05, **P < 0.01

### Sensitivity analyses

Results of the sensitivity analyses for participants without asthma were substantially consistent with primary findings. In the analyses that defined accelerated cognitive decline as a reduction in cognitive scores ≥1 SD standardized over the follow-up time and baseline age, the inverse association was only found in global cognition in males. The E-value suggested that the OR of the association between an unmeasured confounder and baseline PEF and global cognitive decline would need to be at least 1.251 for man and 1.129 for woman for each association to explain away the associations observed in our study. For the competing risk analysis, baseline PEF was found to be inversely associated with global cognition decline in males, which was consistent with the primary analyses (Table 5).

**Table 5:** Adjusted odds ratio of cognitive decline for PEF across all sensitivity analyses

	**Global cognition**		**Mental status**		**Episodic memory**	
		
	**Male**	**Female**	**Male**	**Female**	**Male**	**Female**
Cognitive change of 1 SD standardized by follow-up time, n (%)						
No decline	4077 (93.21)	3624 (92.92)	4236 (96.84)	3780 (96.92)	4050 (92.59)	3539 (90.74)
Decline	297 (6.79)	276 (7.08)	138 (3.16)	120 (3.08)	324 (7.41)	361 (9.26)
Adjusted odds ratio of follow-up time and age standardized 1 SD cognitive decline per 1-SD higher PEF at baseline^†^						
OR (95%CI)	0.896 (0.813, 0.988)	0.978 (0.885, 1.080)	0.933 (0.845, 1.031)	0.914 (0.822, 1.017)	1.037 (0.935, 1.151)	0.964 (0.873, 1.065)
*P*-value	0.027	0.658	0.172	0.098	0.489	0.472
Adjusted odds ratio of cognitive decline per 1-SD higher PEF at baseline for participants without asthma^†§^						
OR (95%CI)	0.922 (0.854, 0.995)	0.970 (0.899, 1.048)	0.889 (0.825, 0.959)	0.958 (0.887, 1.034)	0.908 (0.841, 0.979)	0.813 (0.845, 0.987)
*P*-value	0.038	0.441	0.002	0.268	0.012	0.021
Hazard ratio of competing risk analysis^¶^						
SHR (95%CI)	0.969 (0.941, 0.998)	0.995 (0.965, 1.025)	0.977 (0.947, 1.008)	1.026 (0.993, 1.059)	1.005 (0.968, 1.044)	0.976 (0.942, 1.012)
*P*-value	0.037	0.723	0.151	0.121	0.778	0.194
E-value of adjusted odds ratio of cognitive decline per 1-SD higher PEF at baseline						
OR (95%CI)	0.921 (0.855, 0.993)	0.974 (0.904, 1.050)	0.889 (0.826, 0.956)	0.975 (0.905, 1.051)	0.907 (0.842, 0.977)	0.915 (0.848, 0.987)
E-value	1.251	1.129	1.314	1.126	1.279	1.263
Lower limits of CI	1.063	1	1.175	1	1.121	1.088

Abbreviations: OR, odds ratio; CI, confidence interval; PEF, peak expiratory flow; SHR, significant hazard ratio.

^†^Adjusted for baseline age, education, marital status, registered residence, height, weight, alcohol use, smoking status, diabetes, hypertension, dyslipidemia, social activity, health insurance status, follow-up time and baseline cognitive status.

^§^The number of participants without asthma was 7297 (3756 men and 3541 women).

^¶^Adjusted for baseline age, education, marital status, registered residence, height, weight, alcohol use, smoking status, diabetes, hypertension, dyslipidemia, social activity, health insurance status and baseline cognitive status.

## Discussion

The present study showed that impacts of the baseline PEF on the later cognitive decline were different between the sexes. Higher baseline PEF was significantly inversely associated with the absolute value of cognitive decline. The inverse association between baseline PEF and cognitive decline was stronger in men and individuals >60 years.

In this study, the PEF was lower than expected values [35]. This may be due to the inclusion of patients with asthma or chronic lung disease, as well as the lacking of experience for some subjects to use the instrument. Nevertheless, the PEF was measured using equipment of the same model and the operators were trained uniformly in each follow-up, which ensured comparability of PEF variation between subjects. And previous studies have shown that Chinese population with an age range or average age close to this study has a similar PEF to our baseline value [28, 36]. On this basis, our study found that the baseline PEF level was significantly inversely associated with the decline in global cognition, mental status, and episodic memory in males, and in episodic memory in females. Consistent with previous longitudinal studies that supported baseline associations between pulmonary function and cognition [6, 7, 9, 10, 37], the finding indicated that PEF might be a prominent risk factor or predictor of cognitive impairment in older adults (Supplementary Table 2). The hypothesized relationship has some possible biological mechanisms. Poor lung function would initiate an inflammatory response [38, 39], which may evolve into low-grade systemic inflammation and cause an adverse vascular response and additional oxidative stress [40]. Inflammation and oxidative stress in the central nervous system are associated with the pathogenesis of dementia [41]. Recently, two studies that explored alterations in functional brain areas following the cognitive decline in patients with COPD, indicated the associations, especially in the situational memory part, from a pathogenetic perspective [42, 43].

Nevertheless, current evidence may have been underpowered to draw a valid conclusion as associations between changes in pulmonary function and cognitive function differed widely across studies on middle-aged and older people. Our results showed that the significant inverse associations between positive PEF variation and the rates of 4-year decline in cognitive functions were only found in men. However, another study focusing on men reported that annual rate of decline in FEV_1_ was not consistent with changes in cognitive performance [6]. Notably, there were some studies that reported remarkable findings based on all participants including both males and females [44, 45]. MacDonald and colleagues [9] reported that PEF decline was significantly associated with decline in computation span, fact recall, and vocabulary. Emery and colleague [46] reported that decline in FEV_1_ predicted decline on fluid cognitive function, but did not predict decline in the memory function, which differed from our findings. Additionally, there was a recent study conducted by Shang using CHARLS data, finding that the increment of pulmonary function associated with all cognitive functions by repeated-measures analysis of multiple time points [37]. The inexplicit results across studies are likely due, in part, to the variability in the analysis methods and operationalization, especially the measures of pulmonary function (e.g., PEF vs. FEV_1_ vs. forced vital capacity (FVC)) and cognitive function, limiting the ability to make informed comparisons. Moreover, currently used indicators were relatively single, multiple criteria to comprehensively reflect the pulmonary function were needed in future studies.

The difference in sex of an association between pulmonary function and cognition analyzed with binary logistic regression models in this study was somewhat surprising, as results from another study using the same database draw an inverse conclusion [37]. Shang’s study showed that the association between PEF and global cognitive score, over time, was stronger in women and never smokers. The findings in women were explained with the fact that women are more sensitive to physiological changes related to decreased pulmonary function than men [47, 48]. However, our findings that the inverse associations between positive PEF variation and decline in cognitive functions were only found in men, can be possibly explained with sharper decreasing PEF from baseline in men than that reported by Shang [37]. This shows that the results of these two studies were accordant and complementary, and the discrepancy might be attributed to different analytical methods applied. Notably, the difference between sex was equivocal as significant findings were still scarce, and covariates known to be important were not consistently considered across studies [11].

We found that the baseline PEF was more likely to be inversely associated with cognitive decline in men >60 years. Previous studies have found that the rate of decline in lung function levels accelerates significantly with increasing age in older adults [49] due to age-related changes in thoracic posture and general stiffening of the ribs [50] and the aging of the airway epithelial and cartilaginous tissues. The progressive decrease in pulmonary function and the state of chronic long-term hypoxia in older adults may be the most important cause of reduced cognitive function. Regarding the effect of smoking on cognitive decline, numerous studies have confirmed the independent risk of smoking for cognitive dysfunction [51–53]. In this study, we found it interesting that the degree of reduction in PEF appeared to be more associated with global cognitive decline in never smokers than in men with a prior smoking history or current smoking, yet with no significant difference between the two groups. The results were consistent with the study conducted in men [6], reporting that the association between average FEV_1_ and cognitive decline was generally much greater in never smokers. However, the inapparent results in this study may be attributed partially to the short follow-up period of the CHARLS, and the data available was inadequate to find an effect of smoking history on the relationship between reduced PEF and global cognitive decline. Besides, the inverse association of baseline PEF and cognitive decline was only found in global cognition in sensitivity analyses for cognitive decline ≥1 SD, which was partially consistent with the primary analyses. Although the definition of clinically significant cognitive impairment is standard for older adults, the prevalence of cognitive impairment in middle-aged adults was relatively low, and the characterization of cognitive change is not as well defined in middle-aged adults [34]. By considering the above points, we defined cognitive decline as a change of cognitive score <0, so as to observe both the mild and significant cognitive decrease in middle-aged and older adults. Nevertheless, since the definition of outcome affects study results, the findings need to be interpreted and generalized with caution regardless of how cognitive decline was defined.

### Limitations

There are a few limitations to this study. First, there might be potential selection biases in the study, as individuals included were healthier than those excluded, making it easier to characterize the associations between PEF and cognitive decline. Second, air pollution and other factors that might affect the pulmonary function and therefore influence the association observed were not controlled. However, available covariates reported to be important were included based on previous studies, and the point estimate of E-value seemed moderately robust. Additionally, we found that the indicators used to reflect pulmonary function and the findings of current longitudinal studies varied greatly, hindering the evidence integration of the actual relationship between pulmonary function and cognitive decline. Therefore, we suggest more comprehensive and accurate pulmonary function detecting, longer follow-up and conducting relevant mechanistic studies in future research to clarify the association.

## Conclusions

Pulmonary function correlates inversely with the absolute value of cognitive decline in middle-aged and older people, especially in males, indicating that early efforts to maintain pulmonary function may have a positive influence on cognition later in life. However, additional studies investigating early and long-term PEF changes are needed to provide further solid evidence.

## Supplementary information

**Additional file 1:** **Supplementary Table 1** Comparison of baseline characteristics between included subjects and excluded individuals. **Supplementary Table 2** The basic information of studies focused on pulmonary function-cognition relationships and related mechanism.

## Declarations

### Data availability

The datasets that support the findings of the current study are available from the corresponding author on reasonable request. The data are publicly available and can be downloaded at http://charls.pku.edu.cn/index/zh-cn.html.
