# Supplementary material for: Associations between pulmonary function and cognitive decline in the middle-aged and older adults: evidence from the China Health and Retirement Longitudinal Study
Source: Environ Health Prev Med. 2022 Dec 16;27:48. doi: 10.1265/ehpm.22-00158 (PMC9792564; doi:10.1265/ehpm.22-00158)
Supplement: Supplementary file 1 — Additional file 1: Supplementary Table 1 Comparison of baseline characteristics between included subjects and excluded individuals. Supplementary Table 2 The basic information of studies focused on pulmonary function-cognition relationships and related mechanism. [file ehpm-27-048-s001.docx]

**Associations between pulmonary function and cognitive decline in the middle-aged and older adults: evidence from the China Health and Retirement Longitudinal Study**

**Short title: Pulmonary function and cognitive decline**

Xuefeng Lai^1*^, Jian Sun^2,3,4*^, Bingjie He^1^, Daowei Li^2,3,4^, Shengfeng Wang^1^, Siyan Zhan^1,5,6^

**Author affiliations:**

1. Department of Epidemiology and Biostatistics, School of Public Health, Peking University, 38 Xueyuan Road, Haidian District, Beijing 100191, China

2. Department of Pulmonary and Critical Care Medicine, Shandong Provincial Hospital, Cheeloo College of Medicine, Shandong University, Jinan 250021, China

3. Department of Pulmonary and Critical Care Medicine, Shandong Provincial Hospital affiliated to Shandong First Medical University, Jinan 250021, China

4. Shandong Key Laboratory of Infectious Respiratory Disease, Jinan 250021, China

5. Research Center of Clinical Epidemiology, Peking University Third Hospital, 49 Huayuan North Road, Haidian District, Beijing 100191, China

6. Center for Intelligent Public Health, Institute for Artificial Intelligence, Peking University, Beijing 100191, China

^*^Xuefeng Lai, and Jian Sun contributed equally to this manuscript.

**Correspondence to:**

Shengfeng Wang (email: shengfeng1984@126.com; telephone: 86-10-82801528; mailing address: 38 Xueyuan Road, Haidian District, Beijing 100191, China)

Siyan Zhan (email: siyan-zhan@bjmu.edu.cn; telephone: 86-10-82805162; fax: 86-10-82805162; mailing address: 38 Xueyuan Road, Haidian District, Beijing 100191, China)

Daowei Li (email: ldwchina@163.com; telephone: 053168776360; mailing address: 324 Jing 5 road, Huaiyin District, Jinan 250021, China)


$\geq$

**Additional file**

**Supplementary Table 1** Comparison of baseline characteristics between included subjects and excluded individuals

| Characteristics | Male | | *P*-value | Female | | *P*-value |
| --- | --- | --- | --- | --- | --- | --- |
|  | Included, n=4374 | Excluded, n=2562 |  | Included, n=3900 | Excluded, n=3478 |  |
| Age, mean (SD) ^a^ | 58.16 (8.79) | 61.30 (9.63) | < 0.001 | 56.44 (8.41) | 59.85 (9.88) | < 0.001 |
| Urban, n (%) ^b^ | 1661 (37.97) | 980 (38.27) | 0.809 | 1710 (43.85) | 1311 (37.69) | < 0.001 |
| Marriage ^b^ |  |  |  |  |  |  |
| Married, n (%) | 4061 (92.84) | 2254 (88.01) | < 0.001 | 3479 (89.21) | 2849 (81.91) | < 0.001 |
| Divorced, n (%) | 262 (5.99) | 251(9.80) |  | 418 (10.72) | 627 (18.03) |  |
| Single, n (%) | 51 (1.17) | 56 (2.19) |  | 3 (0.08) | 2 (0.06) |  |
| Education, n (%) ^c^ |  |  |  |  |  |  |
| Illiterate | 351 (8.02) | 373 (19.96) | < 0.001 | 1101 (28.23) | 1364 (50.28) | < 0.001 |
| Primary school | 1959 (44.79) | 890 (47.62) |  | 1560 (40.00) | 923 (34.02) |  |
| Middle school | 1301 (29.74) | 383 (20.49) |  | 819 (21.00) | 274 (10.10) |  |
| High school and above | 763 (17.44) | 223 (11.93) |  | 420 (10.77) | 152 (5.60) |  |
| Smoking, n (%) ^d^ |  |  |  |  |  |  |
| Never | 1139 (26.04) | 582 (27.75) | < 0.001 | 3613 (92.64) | 3144 (91.56) | 0.055 |
| Former | 717 (16.39) | 450 (21.46) |  | 73 (1.87) | 97 (2.82) |  |
| Current | 2518 (57.57) | 1065 (50.79) |  | 214 (5.49) | 193 (5.62) |  |
| Alcohol use, n (%) ^e^ |  |  |  |  |  |  |
| Never | 1303 (29.79) | 747 (29.24) | < 0.001 | 3254 (83.44) | 2808 (80.83) | < 0.001 |
| Former | 395 (9.03) | 377 (14.76) |  | 68 (1.74) | 118 (3.40) |  |
| Current | 2676 (61.18) | 1431 (56.00) |  | 578 (14.82) | 548 (15.77) |  |
| Health insurance, n (%) ^f^ | 4148 (94.83) | 2395 (94.29) | 0.335 | 3671 (94.13) | 3221 (93.55) | 0.305 |
| Socially active, n (%) |  |  |  |  |  |  |
| None | 2454 (56.10) | 1536 (59.95) | 0.007 | 2110 (54.10) | 2124 (61.07) | < 0.001 |
| Inactive | 1191 (27.23) | 642 (25.06) |  | 1140 (29.23) | 888 (25.53) |  |
| Active | 729 (16.67) | 384 (14.99) |  | 650 (16.67) | 466 (13.40) |  |
| Body mass index(kg/m^2^), n (%) ^g^ |  |  |  |  |  |  |
| <18.5 | 209 (4.78) | 186 (7.56) | < 0.001 | 163 (4.18) | 240 (7.09) | <0.001 |
| 18.5~23.9 | 2463 (56.31) | 1386 (56.32) |  | 1684 (43.18) | 1549 (45.77) |  |
| 24.0~27.9 | 1291 (29.52) | 649 (26.37) |  | 1403 (35.97) | 1086 (32.09) |  |
| >28.0 | 411 (9.40) | 240 (9.75) |  | 650 (16.67) | 509 (15.04) |  |
| Height (m), mean (SD) ^h^ | 1.65 (0.07) | 1.64 (0.07) | < 0.001 | 1.54 (0.06) | 1.53 (0.07) | <0.001 |
| Weight (kg), mean (SD) ^i^ | 63.33 (10.98) | 61.83 (11.59) | < 0.001 | 57.90 (10.14) | 55.99 (10.97) | <0.001 |
| Diabetes, n (%) ^j^ | 496 (11.34) | 283 (11.12) | 0.700 | 463 (11.87) | 454 (13.13) | 0.104 |
| Hypertension, n (%) ^k^ | 1535 (35.09) | 1103 (43.20) | < 0.001 | 1379 (35.36) | 1512 (43.54) | < 0.001 |
| Dyslipidemia, n (%) ^l^ | 1120 (25.61) | 581 (22.92) | 0.012 | 1052 (26.97) | 831 (24.19) | 0.006 |
| PEF(L/min), mean (SD) | 327.57 (125.98) | 290.88 (126.53) | < 0.001 | 240.22 (87.87) | 215.42 (87.63) | < 0.001 |
| Global cognition, mean (SD) | 12.46 (3.04) | 10.64 (4.21) | < 0.001 | 11.51 (3.38) | 8.76 (4.51) | < 0.001 |
| Mental status, mean (SD) | 8.59 (2.27) | 7.30 (3.11) | < 0.001 | 7.57 (2.57) | 5.60 (3.35) | < 0.001 |
| Episodic memory, mean (SD) | 3.87 (1.55) | 3.34 (1.73) | < 0.001 | 3.94 (1.64) | 3.16 (1.77) | < 0.001 |

Description of data: Comparison of baseline characteristics between subjects included in final analyses and individuals excluded due to incomplete information of covariates, severe diseases or conditions, cognition score ≤5 and lost follow-up cognitive score.

^a^ 94 missing; ^b^ 1 missing for excluded group; ^c^ 1458 missing for excluded group; ^d^ 509 missing for excluded group; ^e^ 11 missing for excluded group; ^f^ 57 missing for excluded group; ^g^ 195 missing for excluded group; ^h^ 122 missing for excluded group; ^I^ 126 missing for excluded group; ^j^ 36 missing for excluded group; ^k^ 14 missing for excluded group; ^l^ 70 missing for excluded group.

**Supplementary Table 2** The basic information of studies focused on pulmonary function-cognition relationships and related mechanism

| **Longitudinal studies focused on pulmonary function-cognition relationships** | | | | |
| --- | --- | --- | --- | --- |
| First author, year | Sample size | Population, baseline age, % male | Pulmonary measures | Main findings |
| Charles F. Emery, 1998 | 444 | Twins, 40-84 years, 40% | FEV_1_ | Baseline FEV_1_ predicted performance in processing speed and spatial ability measures at baseline, but did not predict change in any of the cognitive outcomes. |
| Charles F. Emery, 2012 | 832 | Twins, 50-85 years, 41% | FEV_1_, FVC | Declines in pulmonary function leads to subsequent decline on cognitive tasks reflecting spatial performance and processing speed, common indicators of fluid cognitive abilities. There was no influence of pulmonary function on memory performance |
| Richards, 2005 | 3035 | Population, 43 years, 49.8% | FEV_1_ | Baseline FEV_1_ was associated with processing speed at baseline and slower decline in processing speed over time. No association between FEV_1_and memory or verbal ability |
| Starr, 2007 | 298 | Population, 64 years, 47% | FEV_1_, PEF, FVC | Higher PEF was associated with better performance on all cognitive tests except Auditory Verbal Learning |
| Aiken-Morgan, 2018 | 407 | Population, 48-95 years, 22.4% | PEF | Better lung function at baseline was significantly associated with cognitive stability over 3 years |
| Infurna, 2013 | 4177 | Population, 66.75 years, 41% | FEV_1_ | FEV_1_ at baseline was significantly associated with 4-year change in memory; better pulmonary function predicted less memory decline |
| Koster, 2005 | 2574 | Population, 70-79 years, - | FEV_1_, FVC | Low FEV_1_at baseline was significantly associated with 4-year decline in cognitive function |
| Pathan, 2011 | 10975 | Population, 45-64 years, - | FEV_1_, FVC | Reduced FEV_1_ or FVC at baseline was significantly associated with worse cognitive function at baseline, and higher risk of dementia at follow-up. No association was observed between reduced FEV_1_ or FVC and cognitive decline |
| Swan, 1992 | 792 | Twins, 57.1/56.3 years, | FEV_1_, FVC | Cognitive decline in twin pairs measured by the digit symbol substitution test had significantly poorer lung function than twin pairs with no cognitive decline |
| Vidal, 2013 | 5764 | Population, 52 years, 33% | FEV_1_/height^2^ | Lower FEV_1_/height^2^ at mid-life were more likely to have lower cognitive test scores or to develop MCI or dementia 23 years later |
| Whitfield, 1997 | 224 | Population, 70-79 years, 40% | PEF | Baseline PEF was significantly associated with cognitive performance. PEF significantly predicted cognitive decline between intervals |
| Shang XW, 2021 | 8264 | Population, 50-95 years, 50% | PEF | High baseline pulmonary function was associated with a lower rate of cognitive decline independent of confounders, especially in women, never smokers, or low-educated individuals |
| **Studies on related mechanism** | | | | |
| First author, year | Sample size | Population | Age (mean/range) | Main findings |
| Lv Z, 2021 | 62 | Patients with COPD vs. healthy subjects | COPD: 72 years;  Control: 71 years | COPD patients exhibited decreased local spontaneous activity in the left basal ganglia. There were significant relationships between static and dynamic local spontaneous activities and clinical characteristics (both pulmonary function and semantic-memory performance). |
| James W. Dodd, 2013 | 110 | outpatients with COPD vs. healthy control subjects | COPD: 70, 69 years;  Control: 65 years | patients with COPD who are judged medically fit for discharge following an exacerbation have significant deficits in cognitive function including processing speed and the loss in the pathologic range |
| J. NOBLE, 1993 | 24 | Healthy males | - | Hypoxaemia was shown to cause a significant impairment of simple unprepared reaction time |
| J. van der Post, 2002 | 12 | Healthy volunteers | 19-27 years | There were a number of effects of hypoxemia on CNS function |
| Lies Lahousse, 2013 | 810 | Patients with COPD and normal subjects | ≥55 years | COPD is associated with a higher prevalence of cerebral microbleeds, a marker of cerebral small-vessel disease determined by MRI. COPD is an independent risk factor to develop deep or infratentorial cerebral microbleeds. |
| P.S. Sachdev, 2006 | 469 | Population | 60-64 years | Subjects with low pulmonary function had greater brain atrophy, in particular on a measure of subcortical atrophy |
| Chang-Woo Ryu, 2013 | 31 | Patients with COPD and normal subjects | COPD: Severe-69.8 years; Moderate-65.2 years;  Control: 63.9 years | FA decreased in prefrontal lobes in moderate COPD compared with normal control subjects. Severe COPD showed extensive regions with significantly lower FA and higher trace in gray and white matter than control groups |
